# Secondary rhinoplasty

**Published:** 2008-10

**Authors:** Gaith Shubailat

**Affiliations:** American Board of Plastic Surgery

## INTRODUCTION

It is important to remember that the nose is a functional organ and not just a cosmetic facial structure; knowledge of its anatomy and physiology is therefore crucial for surgeons performing rhinoplasty. The two objectives of performing any rhinoplasty are to improve the aesthetics and to maintain or restore its normal function. Lack of appreciation of this tenet leads patients to suffer permanent impairment of nasal function.

In a typical busy rhinoplasty practice 25-30% of rhinoplastys are secondary surgery on patients who have had one or multiple previous operations by other surgeons. This includes both oto-rhino-laryngologists as well as plastic surgeons. In the ongoing arguments for and against endonasal or open approaches for primary or secondary rhinoplasty, it became clear to me that two schools of thought developed. Proponents of the endonasal approach like Sheen[1] and Constantian[5] seem to place more emphasis on restoring the shape and balance of the soft tissues by adding cartilage graft fillers. Proponents of the open approach such as Gunter[8] and Gruber[12] felt that reconstructing the perfect skeletal anatomy with both sutures and grafts was more suitable, for which the open approach renders unrivaled operative exposure.

It is only through open rhinoplasty that the author was able to manage and reconstruct 458 post-rhinoplasty deformities, an experience I wish to share in this article. I met the same satisfaction of improved results in 1200 of my other primary rhinoplasty cases.

Physical and radiological examinations can never make the exact diagnosis of the damage that had been sustained in previously operated cases. It is only through the open approach exposure that one can see and define the damage and accordingly reconstruct the deformity in the case on hand. No one case is similar to the other. The literature is rich with elegantly described techniques,[1–11,13–17] but an experienced surgeon will combine techniques, and perhaps add a touch of his/her own to tailor the right operation for each particular situation.

## MATERIALS AND METHODS

In the period from November 1992 to December 2007, a total of 1658 rhinoplasty procedures were performed. Four hundred and fifty eight cases (27%) had had one to nine previous failed operations. Seventy two cases (4.5%) came under the Salvage Rhinoplasty category in whom cantilever osteocartilagenous 10^th^ and 11^th^ rib grafts with micro screw fixation were used with good long-term success rate.[18,19] Autogenous cartilage grafts mostly from the nasal septum and rarely conchal were used in the majority of cases. In 39 patients who refused to have rib or conchal grafts, stored homografts were used, with patients' permission, after excluding hepatitis and HIV in the donor. Alloplastic material was never used.

We used the endonasal approach in over 1600 cases between 1973 and 1992. The advantages of the open technique included a clear well lit exposure of the nasal skeleton that allowed exact evaluation of the damage to the normal anatomy, better exposure of the septum for septoplasty and ease of harvesting cartilage grafts with minimal risk of perforating the septum. The ability to manipulate the upper and lower lateral cartilages under direct vision, precision placement of sutures and grafts lead to much more predictable results. The columellar scar healed well and became invisible in all types of skin, keloid formation did not occur. There was no difference in the resolution of oedema and temporarily impaired tip sensation between our endonasal and open rhinoplasty series.

### Anatomy and physiology

The nose is anatomically subdivided into three compartments; the bony vault, the upper cartilaginous vault, and the lower cartilaginous vault. These have in common a supporting partition, the bony and cartilaginous septum formed by the perpendicular plate of the ethmoid, the vomer, the palatine crest and the quadrangular cartilaginous septum. The bony vault is a pyramidal structure that forms the principle structural base. The lateral walls consist of the frontal processes of the maxilla and the nasal bones with union to the frontal bone in the midline. The middle cartilaginous vault is formed by the upper lateral cartilages (ULC) that are attached to the dorsal edge of the septum like wings at an optimal angle of 15 degrees. The cephalic ends extend deep to the nasal bones for a distance of 11 mm. The caudal edges are connected to the cephalic edges of the lateral crura of the lower lateral cartilages(LLC) with fibro fatty tissue forming an angle of 120 degrees with the septum. The lower cartilaginous vault comprises a pair of lower lateral cartilages, the caudal septal cartilage and nasal spine. The lower lateral cartilage is made of the thin vertical medial crus resting on its foot plate, the curved dome, also known as the middle crus, and the widened lateral crus that attaches to the upper lateral cartilage forming the internal valve. The LLC form the external nasal valve where inspiration in initiated with the nasalis muscle contracting and pulling up the lateral crus. The sub aponeurotic muscular system (SMAS) extends over and covers the nasal skeleton. The surgical dissecting cleavage plane runs deep to SMAS over the skeleton.

### The internal nasal valve

**Figure 1:**
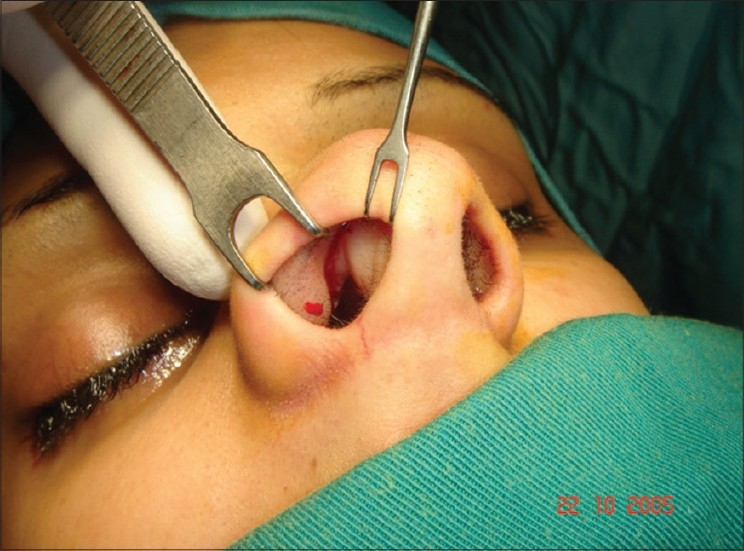
Internal valve. Triangle formed by caudal edge of upper lateral cartilage, septum and floor

**Figure 2:**
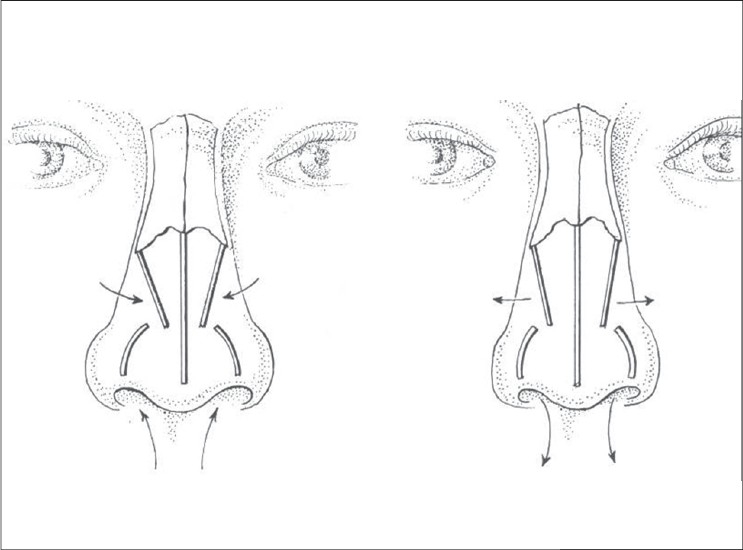
Internal valve action is paradoxical, narrows on inspiration, widens on expiration. Venturi effect

**Figure 3:**
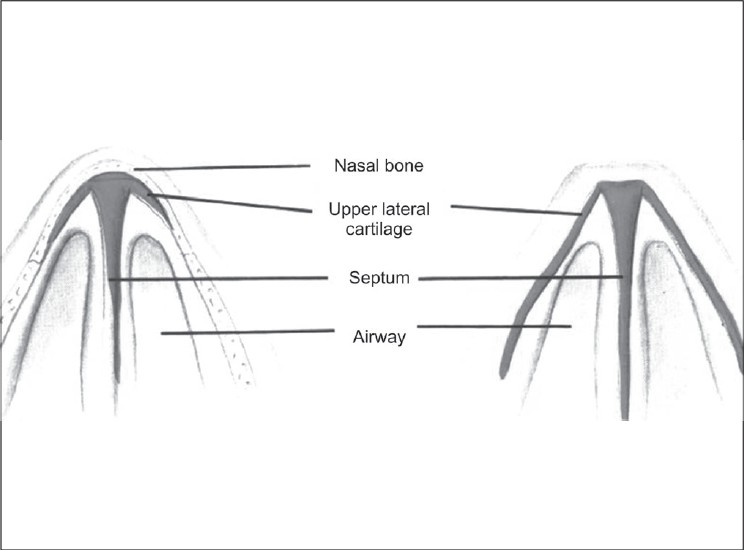
Optimal angle between ULC and dorsal septum is 15 degrees

The internal valve of the nose is the triangle formed by caudal edge of ULC, septum and nasal floor [Figure 1]. Action of the internal valve is paradoxical, narrows on inspiration and widens on expiration giving the Venturi effect [Figure 2]. The optimal angle between the septum and upper lateral cartilage (ULC) is 15 degrees [Figure 3].

**Figure 4:**
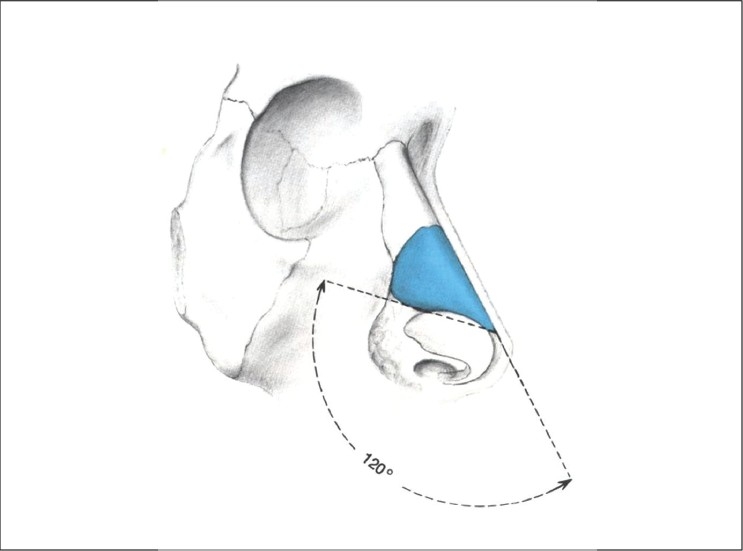
Angle between caudal edge of ULC and septum is 120 degrees

**Figure 5:**
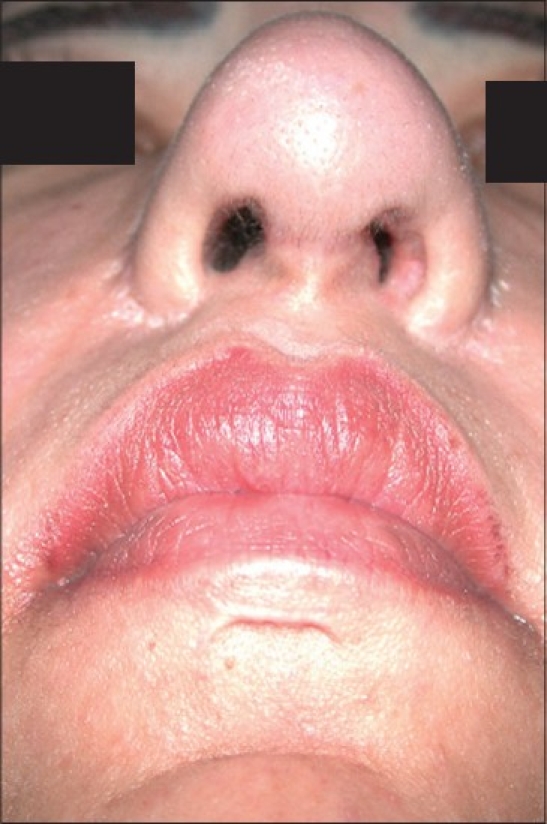
Atresia of internal valve

The normal angle of caudal edge of ULC to septum is 120 degrees and is critical to the functional integrity of the internal valve [Figure 4]. In most secondary cases, breathing difficulties are related to the disruption of the normal anatomical structures forming the internal nasal valve. Atresia can occur in four grades of severity and its correction is very difficult [Figure 5]. Restoring the normal angle of divergence between the dorsal septum and upper lateral cartilage calls for the use of spreader grafts.

### Grafts used

1. Spreader graft is used to restore the normal angle between the septum and upper lateral cartilage.
2. Columellar strut graft is placed vertically between the weak collapsed medial crura to give strong support to the tip.
3. Septal dorsal augmentation grafts are used to raise the dorsum of the nose as in saddle nose where multilayered grafts are usually needed.
4. Tip grafts, shield, umbrella, tombstone etc placed over the domes help define the point of tip differentiation and project the tip.
5. Onlay crushed grafts are used to fill spaces like an open roof, or depressions over the nasal cartilages
6. Rib bone and cartilage grafts are used in salvage cases where the nasal skeleton has been severely damaged and local septal and conchal grafts are inadequate.
7. Homografts are stored in saline impregnated with antibiotic at a temperature of 0-4 degrees. When available, irradiated homografts can be deep frozen. We use these grafts when patients who have had previous resection of the septum refuse to have their ears or rib cage violated.

### Suture techniques

1. Transdomal suture [Figure 6] narrows and elevates the dome or middle crus of the lower lateral cartilage

**Figure 6:**
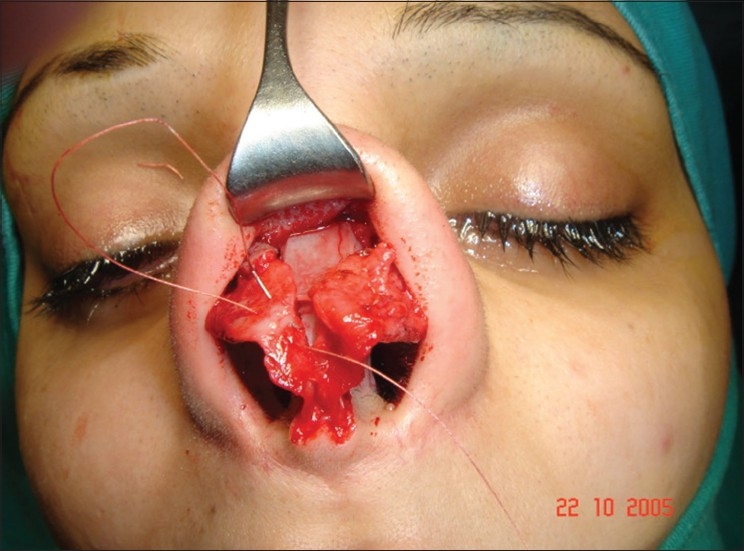
Transdomal suture
2. Interdomal suture approximates the cephalic corners of the narrowed domes to ideally create a 30 degrees angle of convergence by forming the intacrural distance to define to the three point projection of the tip.
3. Columella - septum anchor suture [Figure 7] controls the upward or downward rotation of the tip to get the ideal naso-labial angle which is 90-95 in men and 95-100 degrees in women by European standards.

**Figure 7:**
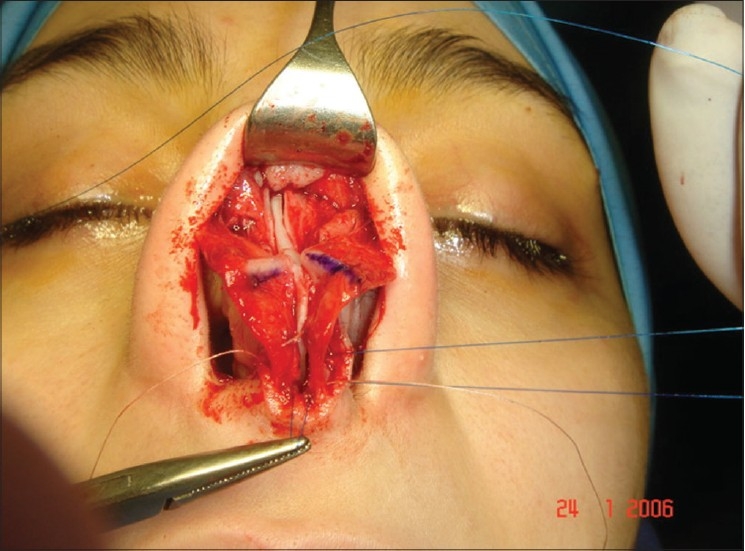
Columellar-septum anchor suture
4. Horizontal mattress suture, converts concavity to convexity and vice versa. Needle enters on one side of the depressed cartilage traverses longitudinally for 6 mm under the concave surface exits then re-enters transversely 3 mm and back to a point 3 mm opposite the entry point. Tightening will straighten the concavity and so vice versa for correcting a convexity [Figure 8].

**Figure 8:**
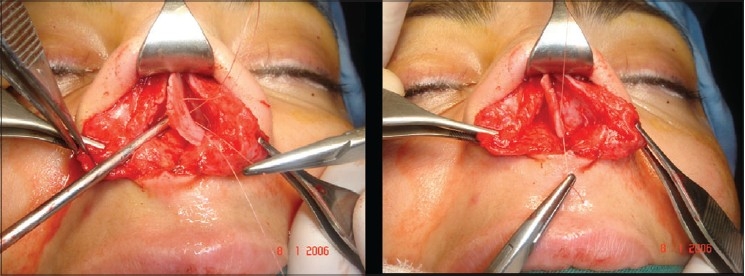
Horizontal mattress suture
5. Differential suture; moves a deviated straight septal cartilage to the midline. It starts through the rigid ULC on the opposite side of the deviation, goes through the deviated septum and opposite LLC then picks a wide bite on the other side and comes back to exit closer to the entry point which on tightening pulls the septum towards the rigid ULC positioning it in the midline. More than one suture may be needed [Figure 9].

**Figure 9:**
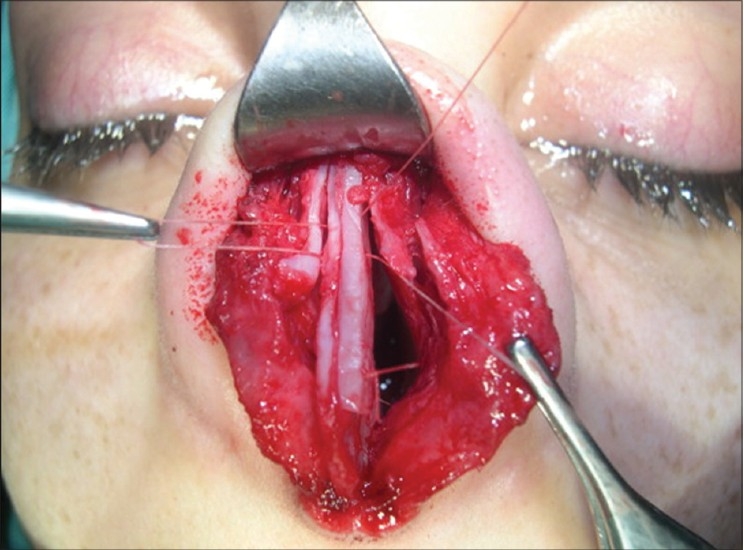
Differential suture

## CASE REPORTS

### Case 1 [Figure 10a,b,c]

**Figure 10a:**
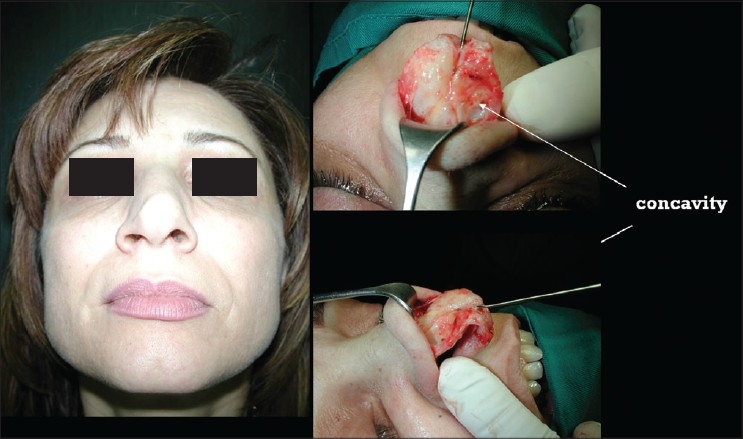
Alar deformity. Concave lateral crus

**Figure 10b:**
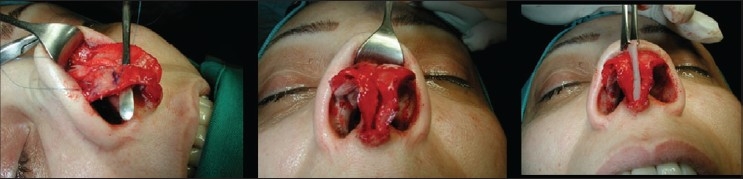
Horizontal mattress suture and columellar strut

**Figure 10c:**
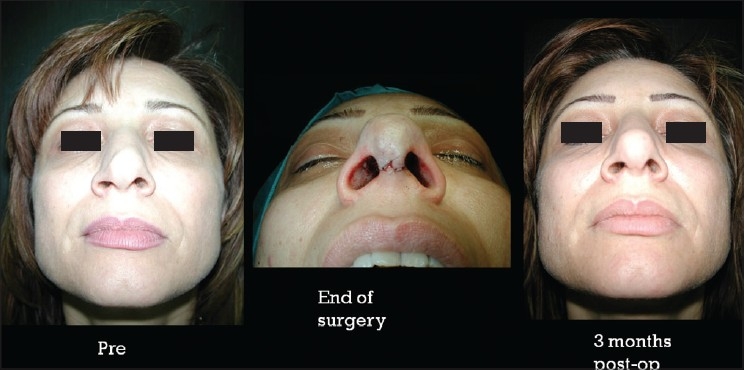
Pre and post-operative views

A 30-year-old patient with concave deformity of the right ala causing a depression was corrected using a horizontal mattress. The columella was reinforced with a strut graft to which the medial crura and domes were approximated

### Case 2 [Figure 11a–e]

**Figure 11a:**
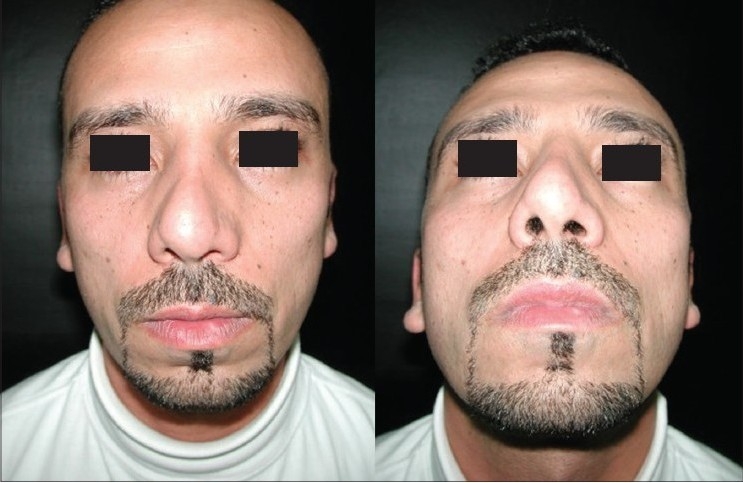
Secondary deformity

**Figure 11b:**
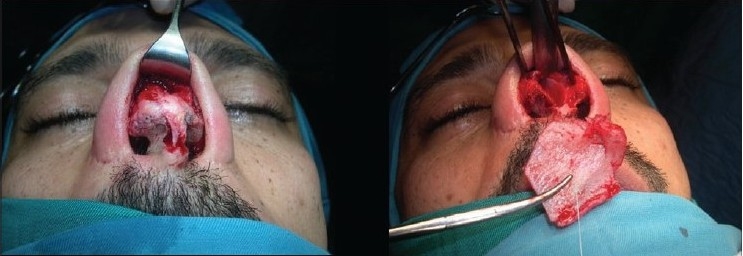
Fibrosis. Extracorporeal septum

**Figure 11c:**
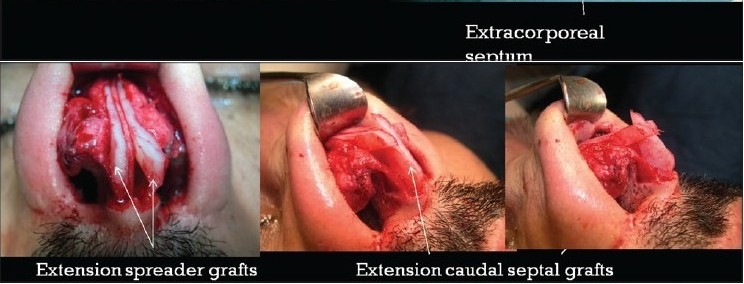
Two extension spreaders holding extension caudal septal graft

**Figure 11d:**
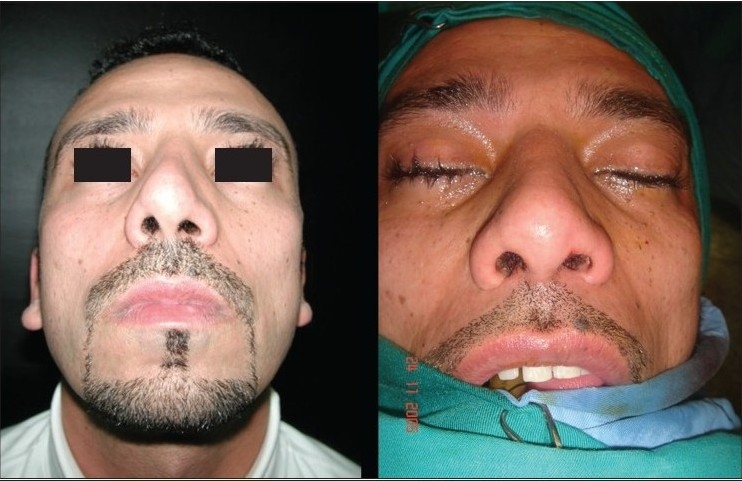
Pre and 20 months post-op. inferior view

**Figure 11e:**
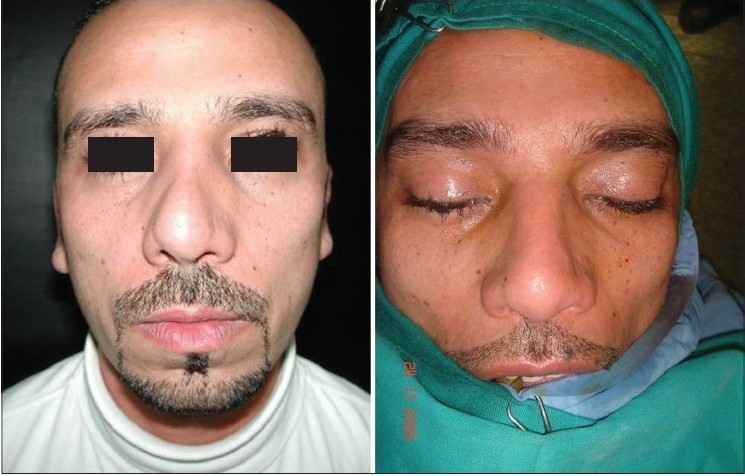
Pre and 20 months post-op frontal view

A 33-year-old male had two rhinoplasty operations resulting in severe destruction of the tissues. The septum had been shortened. Remnant of the septum was delivered and extracorporeal reconstruction of the septum with extension spreader grafts, extension septal graft re-established the normal length of the nose.

The tip was augmented with transverse rectangular umbrella graft and crushed on lay grafts.

### Case 3 [Figure 12a–f]

**Figure 12a:**
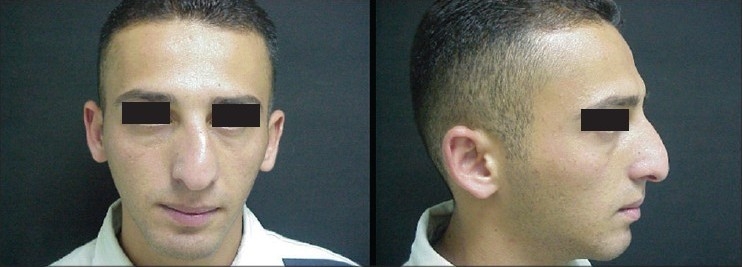
Secondary nasal deformity

**Figure 12b:**
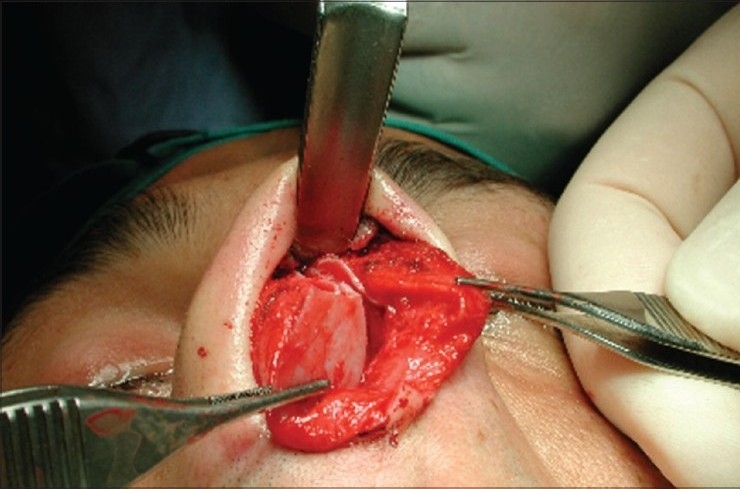
Fracture dislocation of septum

**Figure 12c:**
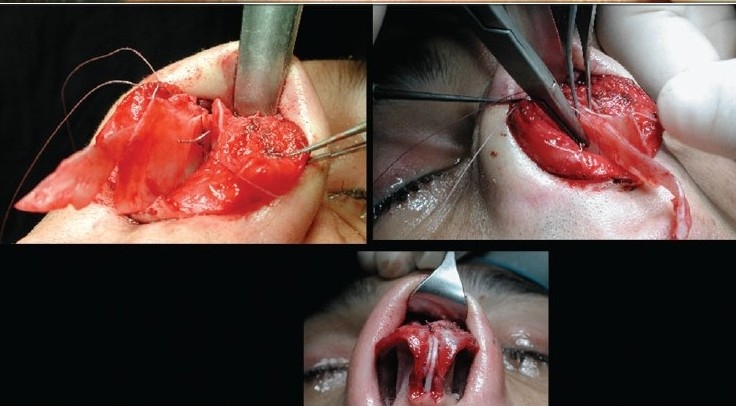
Distal segment advanced and held with buttress extension spreader grafts and medial crura

**Figure 12d:**
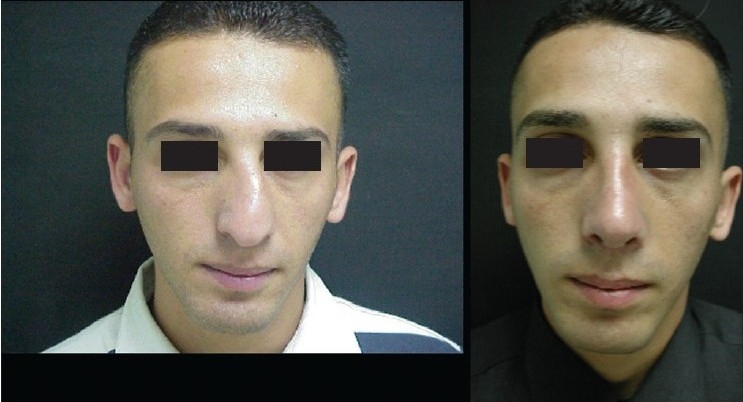
Pre and one year post-op frontal view

**Figure 12e:**
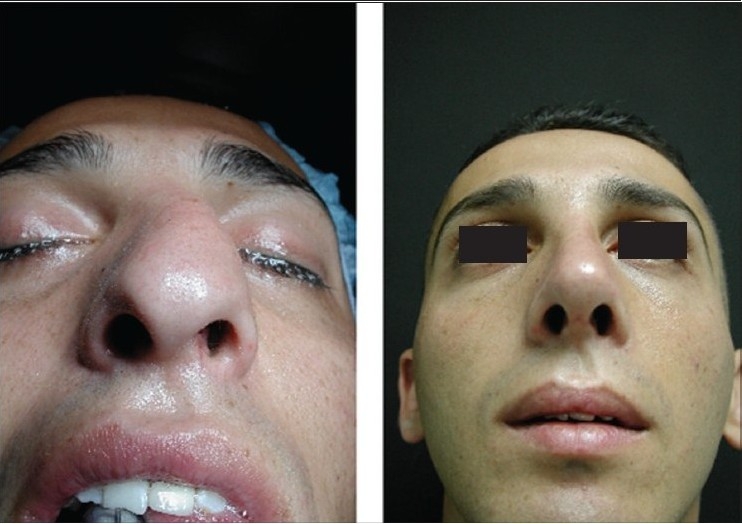
Pre and one year post-op inferior view

**Figure 12f:**
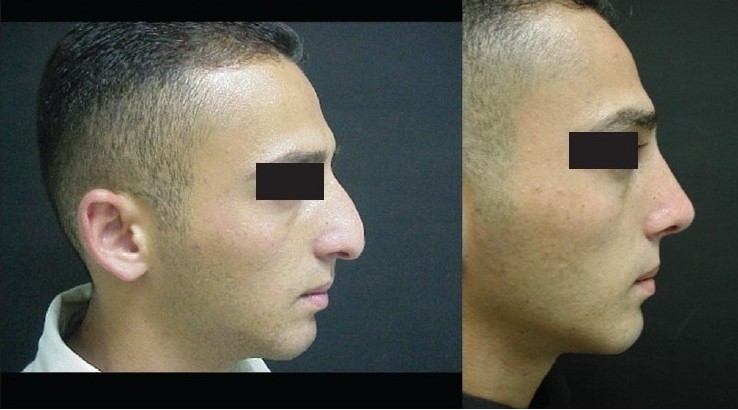
Pre and one year post-op lateral view

A 20-year-old male had post-traumatic secondary nasal deformity. The septum had fractured and was laying transversely causing obstruction and deformity. Distal segment was reduced and held straight in the midline and extended in length with buttress extension spreader grafts to which the medial crura were attached [Figure 12].

### Case 4 [Figure 13a–c]

**Figure 13a:**
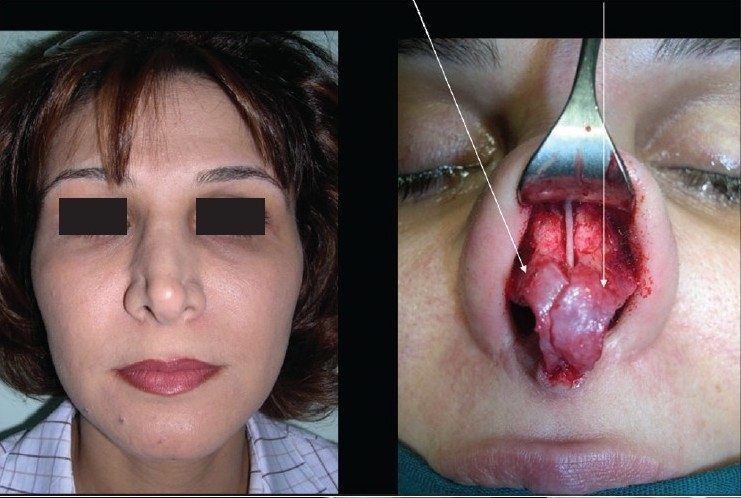
Pinched nose caused by missing lateral crura

**Figure 13b:**
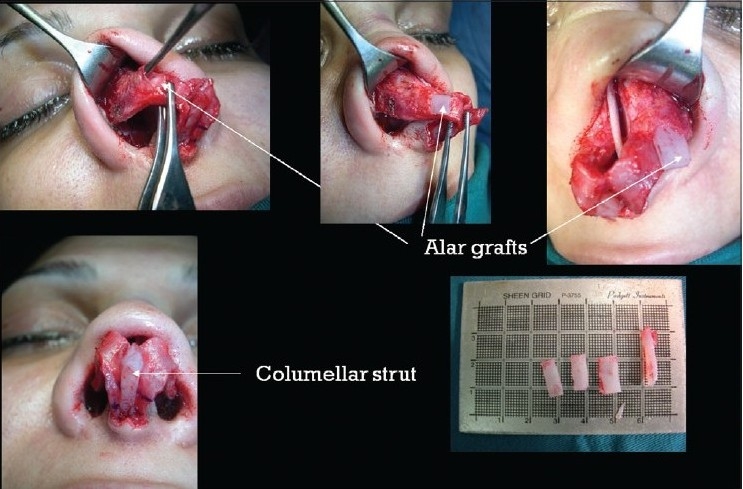
Anatomical Alar grafts and columellar strut

**Figure 13c:**
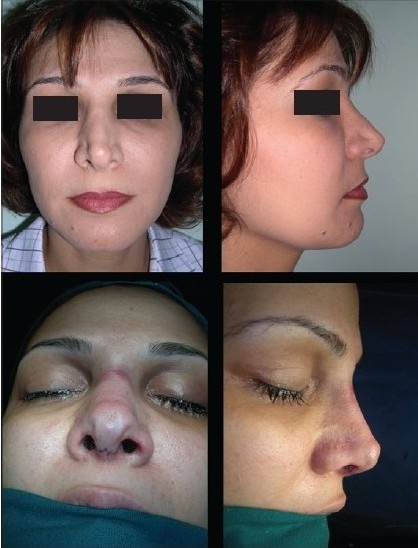
Pre and post-op views

A 29-year-female patient had undergone nasal surgery which resulted in a pinched nose. Exposure of the skeleton with the open transcolumellar technique showed that that the lateral crura were missing having been surgically removed in the previous surgery. This lack of support to the soft rissues of the nasal ala caused collapse and the pinched nose appearance. The missing crura were reconstructed with anatomical alar septal cartilage grafts and a columellar strut to reform and stabilize the triangular nasal base.

### Case 5 [Figure 14a–e]

**Figure 14a:**
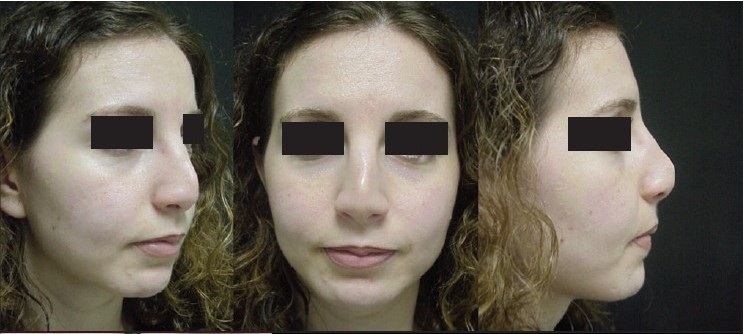
Post rhinoplasty loss of nasal tip

**Figure 14b:**
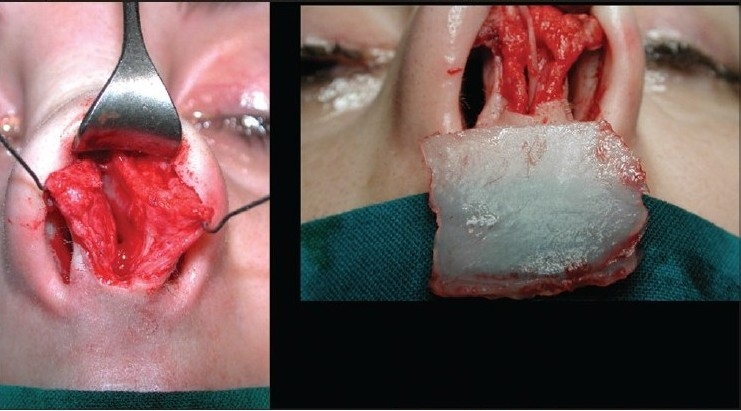
Over-resection of caudal septum. Harvest of septal cartilage for grafting

**Figure 14c:**
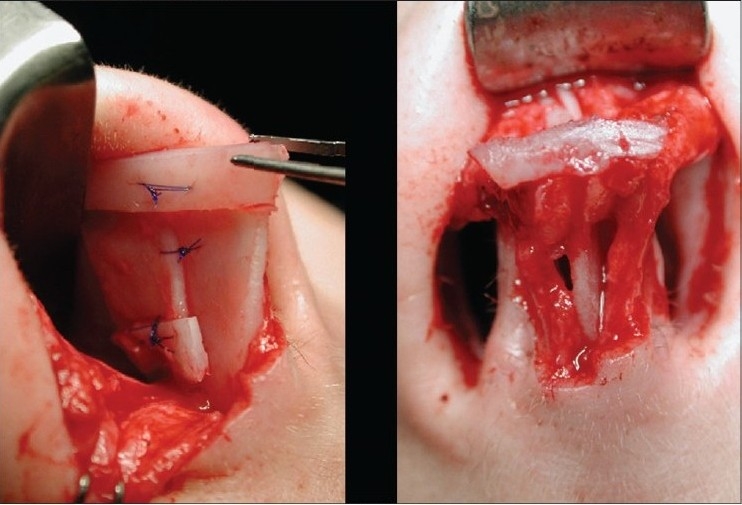
Extension, buttress, spreader and tip grafts

**Figure 14d:**
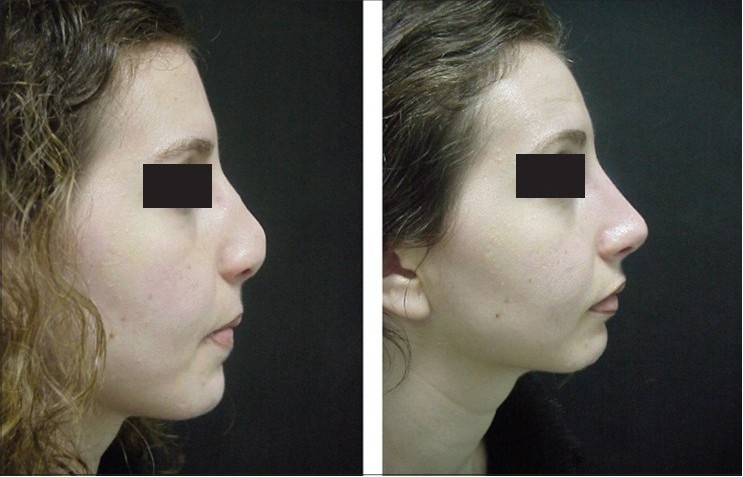
Pre and 1 year post-op, lateral view

**Figure 14e:**
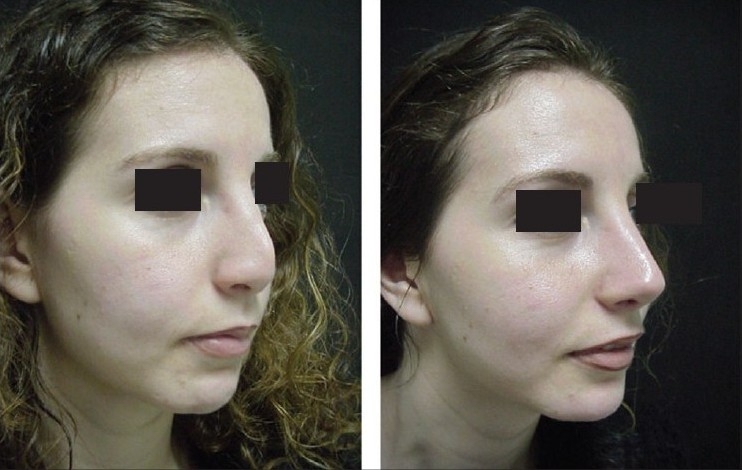
Pre and 1 year post-op, oblique view

A 28-year-female patient presented with short stubby nasal tip after rhinoplasty. Exploration revealed an over resected caudal septum and weak deformed lower lateral cartilages. A large piece of septum was harvested and the cartilaginous framework was reconstructed with an extension septal graft held between two spreader grafts and a basal buttress graft to which the medial crura were attached. The tip was augmented with a transverse tip graft.

## CONCLUSION

Correction of nasal deformities secondary to multiple operations and trauma is a wide and complicated subject. To approach the difficult problem, the surgeon must be in total command of the situation with good knowledge of the anatomy and physiology. In my opinion, only an open transcolumellar approach will allow one to properly diagnose the severe pathology. Reconstructing the anatomy with multiple techniques described by so many good surgeons is superior to all other camouflage operations performed through a limited, constricted and dark field of the endonasal approach. If the open approach exposure is done along the correct lines and planes, there will be no significant observable scarring and deformity of the columella in any type of skin. The best material to use is autogenous cartilage and bone.
